# Validation of the Revised Screening Scale for Pedophilic Interests (SSPI-2) in Portugal

**DOI:** 10.1177/10790632241268502

**Published:** 2024-07-30

**Authors:** Cláudia Gouveia, Marta Sousa, Olga Cunha, Michael Seto, Andreia de Castro-Rodrigues, Rui Abrunhosa Gonçalves

**Affiliations:** 1 Psychology Research Center (CIPSI), School of Psychology, 56059 University of Minho, Braga, Portugal; 2 HEI-Lab: Digital Human-Environment Interaction Lab, 386292 Universidade Lusófona Do Porto, Porto, Portugal; 3 Institute of Mental Health Research, 97559 Royal Ottawa Health Care Group, Brockville, ON, Canada; 4 56068 ISPA – William James Center for Research, Lisbon, Portugal

**Keywords:** child sexual abuse, pedophilia, assessment, atypical sexual interests

## Abstract

The accurate assessment of pedophilic sexual interests is crucial for the treatment and management of individuals who have sexually offended children. This study aimed to validate the Revised Screening Scale for Pedophilic Interests (SSPI-2) in a Portuguese sample of 170 men convicted of sexual offenses against children, 104 serving sentences in the community, and 66 in prison. The findings indicated that SSPI-2 demonstrated good convergent validity, as evidenced by its significant and positive associations with the “sexual deviance” item of SVR-20, the number of previous convictions for sexual crimes against children, and having 3 or more child victims, which is associated with high sensitivity and specificity in distinguishing men who show greater sexual arousal to children than to adults. Furthermore, the SSPI-2 exhibited good divergent validity, with no significant correlations observed with a self-report measure of psychopathy or with a nonsexual criminal history.

## Introduction

Pedophilia is clinically defined as a sexual attraction to prepubescent children (usually age 13 or younger), as reflected in sexual thoughts, fantasies, urges, sexual arousal, or behavior, persisting for at least six months (American Psychiatric Association, 2022). Pedophilia is a robust and important risk factor for sexual recidivism among those who have sexually offended, and thus it is important to assess for sexual attraction to prepubescent children (Hanson & Bussiére, 1998; Hanson & Morton-Bourgon, 2005; Seto, 2018).

There are different ways to assess pedophilia and diagnose pedophilic disorder when someone with pedophilia is experiencing clinically significant distress or impairment as a result of their sexual attraction to prepubescent children. The most straightforward is through self-report, either by interviews or administering questionnaires (see Seto, 2018). However, self-report is vulnerable because of the stigma associated with pedophilia, where individuals may minimize or deny their sexual attraction to prepubescent children, especially when they are facing legal or social consequences from admitting their attraction (such as criminal prosecution).

Another assessment method for men involves the phallometric measurement of genital sexual arousal during the presentation of auditory or visual sexual stimuli depicting people in different age categories (see McPhail et al., 2019, for a systematic review). Sexual arousal to children, relative to adults, consistently distinguishes men who have sexually offended against children from other men and is a strong predictor of sexual recidivism (Hanson & Bussiére, 1998; Hanson & Morton-Bourgon, 2005). Moreover, Blanchard et al. (2001) found that phallometric testing has good specificity and sensitivity when assessing men who have sexually offended against children, where men who have three or more child victims are likely to show greater sexual arousal to children than to adults. However, phallometric testing is expensive, perceived as intrusive, and ethical concerns (e.g., the use of sexually explicit stimuli depicting children) have been raised. In addition, phallometric testing is often unavailable, or individuals may refuse to participate in this testing.

Another objective assessment approach is based on relative viewing time (VT), based on the finding that individuals with pedophilia direct more attention to sexual stimuli involving children than to other sexual stimuli (Fromberger et al., 2012). In most cases, the VT measurement is combined with a measure of self-reported sexual attraction. However, only one study so far has demonstrated that viewing time measures of sexual interest in children have predictive validity (Gray et al., 2015). Yet another way to assess pedophilia comes from eye-tracking research (Fromberger et al., 2012; Godet & Niveau, 2021). The measurement of eye movements offers one possibility to directly explore attentional processes and studies have revealed that individuals with pedophilic disorder show a significant shorter fixation latencies and longer relative fixation times for children’s stimuli, but there are too few studies about its reliability and validity (Fromberger et al., 2012; Godet & Niveau, 2021).

Yet another source of information that can be useful in the ascertainment of pedophilia and diagnosis of pedophilic disorder relies on behavioral correlates of pedophilia among men who have sexually offended against children. Seto and Lalumière (2001) developed the Screening Scale for Pedophilic Interests (SSPI) as a proxy measure for sexual arousal to children when phallometric test results were unavailable, for example, when labs are not accessible or the individual refuses to participate in the testing. Items were selected to be easy to score and objective. Seto and Lalumière (2001) found four behavioral correlates were significantly associated with greater sexual arousal to children in a sample of over 1000 men who had sexually offended against children: having boy victims; having more than one child victim; having a child victim under age 12; and having an extrafamilial child victim. Scores on the SSPI can range from zero to 5, with a point for every item that was present, except for having a boy victim, which receives 2 points. The association between SSPI score and pedophilia has been replicated in different settings and countries (e.g., Banse et al., 2010; Moulden et al., 2009). The SSPI has also been found to predict sexual recidivism among adult offenders with child victims in several studies (Helmus et al., 2015; Seto et al., 2004; but not Canales et al., 2009).

Recently, Seto et al. (2017a) revised the SSPI (SSPI-2) by using unitary weighting for all the items and adding a fifth item regarding child sexual exploitation material (legally referred to as child pornography) offending, given subsequent research showing child sexual exploitation material offending is also strongly correlated with sexual arousal to children (Blanchard et al., 2007; Seto et al., 2006). Scores on the SSPI-2 can again range from zero to 5, and these scores were significantly associated with admission of sexual interest to children and with phallometrically-assessed sexual arousal to children in a large Canadian sample of 1900 men (Seto et al., 2017a). In a companion study, Seto et al. (2017b) found that SSPI-2 scores were significantly associated with new sexual rearrests within five years in a large and independent sample of men convicted of sexual offending against children in the state of New York. This positive association between SSPI-2 and sexual recidivism was also reported by Faitakis et al. (2023) in a study conducted with 626 men referred for a sexological assessment because of sexual offending against children. More recent research suggests the SSPI-2 can be better described as a measure of pedohebephilia (pedophilia as well as hebephilia, a sexual attraction to pubescent children) than pedophilia because of how it correlates with victim ages and with phallometric test response patterns (Stephens et al., 2019).

Seto (2008) presented a conceptual framework delineating two distinct trajectories in sexual offending against children, with one path tied to antisociality (exemplified by psychopathy) and the other path associated with atypical sexual interests, especially pedophilia. Studies typically find weak or no significant associations between psychopathy and pedophilia among individuals who have sexually offended against children (Bommel et al., 2018; Firestone et al., 2000; Kingston et al., 2007; Serin et al., 1994). Of these studies, two of them (Eher et al., 2015; Kingston et al., 2007) used the SSPI for the identification of pedophilic interests, and the correlations between the SSPI and psychopathy were not significant. Additionally, two studies have found that pedophilic individuals who have sexually offended children are lower on psychopathic traits than non-pedophilic individuals who have offended (Boccaccini et al., 2008; Strassberg et al., 2012). Seto et al. (2017b) found that the SSPI-2 was not significantly correlated with antisocial personality or general self-regulation problems and was significantly but weakly correlated with psychopathy scores (*r* = .057).

### Current Study

In Portugal, the assessment of pedophilia and the diagnosis of pedophilic disorder is hindered by a lack of objective assessment methods due to ethical restrictions on the use of phallometric testing and few if any labs that use viewing time measures of sexual interest in children. This means the assessment of pedophilia relies on self-report and the subjective interpretation of these self-reports and sexual offending histories by evaluators. It is extremely important to have less subjective measures that help in this type of assessment, given that individuals with pedophilic interests are at greater risk of sexual recidivism (Aparcero & Rosenfeld, 2022; Seto, 2019) and have different intervention needs than men without pedophilic sexual interests (Leistedt & Thibaut, 2021; Seto, 2008). Therefore, validation of an objective measure of sexual interest in children such as the SSPI-2 would be valuable.

We aimed to validate the SSPI-2 in Portugal in the present study. The first objective of this study was to compare the Portuguese sample with the original study sample reported by Seto et al. (2017a). We then examined the item-total correlations of the SSPI-2 and its convergent validity by exploring its associations with the “sexual deviance” item of SVR-20 (Boer et al., 1997; Portuguese version, Gonçalves & Vieira, 2004; see Methods below). Additionally, we investigated the relationship between SSPI-2 scores and having previous convictions for sexual crimes against children, which indicates a persistent pattern of sexual offending (Helmus et al., 2015; Seto et al., 2004). Furthermore, we examined the association between SSPI-2 scores and the presence of 3 or more child victims, considering previous research by Blanchard et al. (2001) that highlighted the threshold of 3 or more child victims as providing high sensitivity and specificity for phallometric testing in distinguishing those who are likely to be pedophilic by showing greater sexual arousal to children than to adults. Showing an association between SSPI-2 scores and previous convictions for sexual offenses against children or having 3 or more victims is not inherently tautological because the SSPI-2 does not necessarily increase if someone has prior convictions or 3 or more victims. For example, someone who already has multiple child victims in the index offenses would not gain a point for multiple victims, and they may already have a boy victim, victim under age 12, or an unrelated child victim. SSPI-2 scores were calculated based on the total available information, however, so it is not possible to calculate the contribution of any prior sexual offenses and the most recent offense(s).

We also looked at divergent validity by examining whether the SSPI-2 was negatively associated or uncorrelated with psychopathy measured by the Self-Report Psychopathy – Short Form (SRP-SF) (Paulhus et al., 2016; Portuguese version, Seara-Cardoso et al., 2020; see Methods below), and extending this by examining the association of SSPI-2 scores with previous convictions for sexual crimes against adults (Schippers et al., 2023). It is hypothesized that individuals with pedophilic sexual interests would demonstrate lower psychopathy scores as evidenced by studies such as those conducted by Firestone et al. (2000) and Porter et al. (2000). We also expected fewer adult victims and a decreased likelihood of engaging in sexual offenses against adults, based on evidence that interest in children is inversely related to the number of adult partners and involvement in sexual offenses against adults (Schippers et al., 2023). Finally, we would also expect individuals with pedophilic sexual interests (according to the SSPI-2) to be less likely to be involved in prior non-sexual crimes, given previous results that SSPI scores are uncorrelated with nonsexual offense history (Helmus et al., 2015) and sexually offending against children is associated with less extensive nonsexual offending histories (e.g., Baxter et al., 1984; Lussier et al., 2005).

## Method

### Sample

The 170 participants were selected by convenience, for the following criteria: Men aged 18 years or older, involved with the justice system for contact sexual crimes against children under 15 years of age (which can include engaging in sexual activity; compelling or coercing the minor to participate in sexual interactions against his will; or luring or persuading the minor to observe sexual abuse or explicit sexual activities), and convicted (resulting in a suspended sentence, provisional suspension of proceedings, or prison sentence followed by parole). In cases of suspended sentences, the individuals serve their time in the community, but are prohibited from engaging in any criminal activity and must abide by conditions such as restraining orders (e.g., not being allowed to work in professions involving contact with children) and mandatory treatment. In certain cases, the public prosecutor, with judicial authorization, may temporarily suspend criminal proceedings for a certain period. During this period, the same restrictions and conditions are applied.

In Portugal, any sexual behavior with a child under the age of 14 is illegal, resulting in a prison sentence ranging from one to eight years and considered a public offense, requiring reporting by anyone who becomes aware of it. The age at which young individuals can engage in consensual sexual activity with another adolescent is set at 14 years; however, the law provides protection for individuals between the ages of 14 and 16, aiming to prevent situations in which an adult might exploit their lack of experience, categorizing this offense as semi-public (i.e., the complaint must be from the person with standing, usually the victim or their legal representative). According to Portuguese law, the legal age for a minor to engage in relationships with an adult without legal consequences is established at 16 years. This excludes relationships involving financial gain or when the adult is responsible for the child’s education or care.

Participants were an average age of 39.6 years old (*SD* = 14.6), ranging from 18 to 77 years. Most participants (*n* = 160, 94.1%) were of Portuguese nationality and the majority were serving time in the community (*n* = 104; 61.2%). A third (32.9%) had up to primary education (i.e., fourth grade). The majority were married/cohabiting (*n* = 92; 54.1%) and were working at the time of the sexual crime they were sentenced for (*n* = 92; 54.1%) (Table 1).

**Table 1.** Sociodemographic Description of the individuals, Type of Sentence, Description of Victims and Abuse.

Variable	*N* = 170 N (%)
Nationality	
Portuguese	160 (94.1)
Other	10 (5.9)
Academic qualifications	
Up to primary education (4^th^ grade)	56 (32.9)
2^nd^ cycle (5^th^ and 6^th^ grade)	44 (25.9)
3rd Cycle (7^th^ to 9^th^ grade)	37 (21.8)
Secondary education (10^th^ to 12^th^ grade)	25 (14.7)
University education	8 (4.7)
Marital status	
Single	58 (34.1)
Married/cohabiting	92 (54.1)
Divorced	18 (10.6)
Widower	2 (1.2)
Professional situation	
Employed	92 (54.1)
Unemployed	50 (29.4)
Student	6 (3.5)
Retired	20 (11.8)
Prior criminal record	
Without prior crimes	116 (68.2)
Previous sexual offense against adults	2 (1.2)
Previous sexual offense against children	10 (5.9)
Other previous crime	42 (24.7)
Type of penalty	
Prison sentence	66 (38.8)
Community sentence	104 (61.2)
Victim’s age	
Under 12 years old	92 (54.1)
12 and 13 years old	75 (44.1)
14 years old	3 (1.8)
Sex of the victim	
Male	20 (11.8)
Female	140 (82.4)
Both	10 (5.9)
Number of victims	
One victim	128 (75.3)
More than one victim	42 (24.7)
Relationship to victim	
Intrafamilial	67 (39.4)
Extrafamilial	103 (60.6)
Type of crime	
Sexual crimes against children under 15 years old	135 (79.4)
Sexual crimes against children under 15 years old and child sexual exploitation material	35 (20.6)

All individuals were convicted of sexual crimes against children under the age of 15; 20.6% (*n* = 35) had also been convicted of child sexual exploitation material crimes (where Portuguese law prohibits possession, distribution, or production of sexualized images or videos of minors, i.e., below the age of 18; Portuguese Criminal Code^1^). Most of the sample had girl victims (*n* = 140; 82.4%), two-thirds of the sample had offended against unrelated children (*n* = 103; 60.6%), and a quarter (*n* = 42, 24.7%) had multiple child victims. Focusing on victim age and the relevant Portuguese laws, only three individuals had 14-year-old victims; the rest had victims aged 13 or younger. A slight majority had child victims under 12 years old (*n* = 92; 54.1%). Two-thirds of the participants (*n* = 116; 68.2%) had no prior criminal record. Among those who had previous sexual offenses recorded, 10 (5.9%) had prior sexual crimes against children, and only 2 (1.2%) had prior sexual crimes against adults.

### Instruments

#### Sociodemographic Information

The first and second authors independently coded the sociodemographic information from different individuals' files. Sociodemographic information included age, nationality, education, marital status, professional situation.

#### Criminal Variables

The criminal variable information was coded by the first two authors, encompassing specific details about the sexual offense(s) for which the individual was convicted, the type of sentence being served (imprisonment or community-based), the number of victims under 15 years old (indicating the presence of a single victim or multiple victims), the age of the victims (under 12 years old, 12 or 13 years old, 14 years old), the gender of the victims (boy or girl), and the relationship to the victim(s) (extrafamilial or intrafamilial).

We also recorded any convictions prior to the current sentence and coded the following dichotomously (yes/no): no prior convictions; prior convictions for sexual offenses against children; prior convictions for sexual offenses against adults; and prior convictions for nonsexual offenses.

Revised Screening Scale for Pedophilic Interests (SSPI-2; Seto et al., 2017a)

The SSPI-2 includes five items: any boy victims under the age of 15; more than one victim under the age of 15; any victim under the age of 12; any extrafamilial victim under the age of 15; and whether the person has admitted to viewing child sexual exploitation material or been charged with a child sexual exploitation material crime. Each item is scored as absent (score of zero) or present (score of 1), with the total possible score ranging from zero to 5. The higher the score, the more likely someone will show greater sexual arousal to children than to adults in phallometric testing, with a score of 4 or 5 suggesting pedohebephilic sexual interests (the person is more likely than not to show greater sexual arousal to prepubescent or pubescent children). The SSPI-2 is an actuarial tool because it is based on static, historical factors and each score is associated with a probability of showing greater sexual arousal to children than to adults.

The SSPI-2 can be scored with the information from forensic reports about victims of sexual crimes, as well as the individual’s self-reported sexual offending. File data override self-report when the person denies information in the records, but admission of previously unknown child victims or child sexual exploitation material offending are included. In this study, the SSPI-2 was completed at a later stage in the assessment process of the individuals, after the interviews, scoring of the SVR-20 by the researchers, and completion of the self-report questionnaires by the participants. Therefore, it was not possible to access the participants’ self-reported victims, unless it was present in the individual’s court files.

Consequently, only the file data were consulted for its completion, which included information about the individual’s current crime(s) and criminal record. The SSPI-2 scoring was carried out by the first and second authors of the article separately for each individual of the sample. It was scored based on the person’s lifetime history of sexual offenses against children, not only the index offense. Both evaluators coded the SSPI-2 for all participants, with perfect (100%) agreement.

***Sexual Violence Risk - 20*** (SVR-20; Boer et al., 1997; Portuguese version Gonçalves & Vieira, 2004)

SVR-20 is a structured professional judgement tool that systematizes information collected on an individual, identifying problem areas to assess the risk of recidivism in adult male convicted for sexual crimes. The instrument is composed of a set of risk factors for sexual violence, and its completion is performed by professionals in the form of a checklist. The items are answered in relation to the “presence” (S), the “possible presence” (?), “absence” of the risk factor (N), or whether it is “hidden” (i.e., there is no information about it: O). The SVR-20 is divided into three axes: Psychosocial adjustment, Sexual offenses, and Future plans. The results regarding the risk of sexual violence are distinguished between low, medium, and high.

Coders had previous training to carry out the risk assessment and use the SVR-20. The completion of this instrument was always carried out by the first or second author and discussed with the last author of the article. The SVR-20 was completed in accordance with the manual’s instructions. This process involved gathering information from the individual records of each participant and conducting interviews to verify the necessary information for completing the instrument. The “sexual deviance” item was considered as present only if individuals acknowledged, through self-report, being sexually attracted to children or if a diagnosis of pedophilic disorder was documented in the files, specifically within the individual’s clinical history or following evaluation through forensic psychological examination, conducted by a psychologist or psychiatrist. For the “possible presence” classification, we considered the existence of any notes by the current prison psychologist suggesting or indicating pedophilia, but without a formal diagnosis or without self-report from the participant. Due to lack of information, 16 individuals could not be scored on the “sexual deviance” item.

To carry out the ROC curve and logistic regression analyses using the sexual deviance SVR-20 item, this variable was recoded to be dichotomous, so “absent” or “present”. Only individuals who had an associated diagnosis or an objective self-report regarding their sexual interests were coded as “present”. Individuals identified in the item as “possibly present” were coded as “absent”.

In this study, a total of three coders were involved in scoring the SVR-20. The two primary coders were assigned to evaluate different individuals, and then the result was discussed with the third coder, until a consensus was reached. The two primary coders were not involved in the scoring of the SVR-20 for the same individuals, so each SVR-20 was assessed by only two researchers (the coder who conducted the participant interview and the last author of the article).The inter-rater reliability of the SVR-20 has been demonstrated to be moderate to good (e.g., Hart & Boer, 2009), with just two studies (Hildebrand et al., 2004; Sjöstedt & Långström, 2002) demonstrating inter-rater reliability that ranged from low to fair.

***Self-Report Psychopathy – Short Form*** (SRP-SF; Paulhus et al., 2016; Portuguese version Seara-Cardoso et al., 2019)

SRP-SF is a self-report instrument made up of 29 items. The instrument accesses four facets of psychopathy: interpersonal (INT), affective (AFF), lifestyle (LIF), and antisocial (ANT). Except for the ANT subscale, each subscale is composed of seven items, answered on a 5-point Likert scale ranging from 1 (strongly disagree) to 5 (strongly agree). The scores for each facet are obtained by adding up the individual scores of the corresponding items. The INT subscale assesses dissocial characteristics such as pathological lying and manipulation; AFF addresses the affective aspects of psychopathy, such as impaired empathy and lack of guilt and concern for others; LIF relates to impulsive and reckless behaviors; ANT refers to overt antisocial behaviors and includes eight items, but only seven items are considered for modeling purposes. The item “committed a crime” is omitted in justice-involved samples and the item “gang activity” is omitted in community samples due to low variability in these respective groups. Regarding internal consistency, in the Portuguese adaptation, Cronbach’s alpha revealed values ranging from .71 to .87 (Seara-Cardoso et al., 2019). In the present sample, the instrument showed a Cronbach’s alpha of .82 for the total scale; .68 for the INT subscale; .43 for the AFF subscale; .70 for the LIF subscale; and of .71 for the ANT subscale. The alphas for the INT and AFF subscales fell below the acceptable level and were, therefore, excluded from the analyses.

### Procedure

#### Translation of SSPI-2

A team of three psychology researchers fluent in Portuguese and English carried out the translation procedure. One researcher translated the items into Portuguese, and another translated the items back into English. These versions were then discussed, and a consensus translated version was created.

#### Data Collection

The research was approved by the Ethics Committee for Research in Social and Human Sciences of the University of Minho. For collecting data, permission was obtained from the General Directorate of Reintegration and Prison Services – Ministry of Justice (*Direção Geral de Reinserção e Serviços Prisionais – Ministério da Justiça*) and from the Psychology Association of the University of Minho.

The study was performed with the collaboration of four community services in the north of the country and six national prisons. To collect data, institutions were contacted to schedule a meeting to explain the aims of the study and start data collection. In collaboration with the staff, we were able to identify 178 men convicted of sexual crimes against children. The potential participants were informed about the study’s conditions and the confidential and voluntary nature of the study. Among the individuals, eight refused to participate in the study. Those who agreed to participate provided written informed consent and responded to an interview to obtain information regarding the SVR-20 items. As previously mentioned, two coders assessed different sets of participants, with the involvement of a third coder. The scoring was initially conducted by the primary coder, who, after completing their scoring and deciding on the risk to assign to the participant, would meet with the third coder to discuss the assigned risk and the scoring performed. In cases where there was disagreement between the primary coder and the third coder in the coding of the instrument, discussions were held between them until a consensus was reached. The item “sexual deviance” was quoted as present only when individuals admitted through self-report to being sexually attracted towards children or when a diagnosis of pedophilic disorder was mentioned in the procedural data. No compensation or reward was given for participation in the study.

The SSPI-2 was completed after the interview and was coded only using the data present in the individuals' criminal and court records. The files also include self-reported information that was provided in court or in a previous psychological evaluation. These details encompass not only the current offense in question but also any past offenses that may be recorded in the individual’s criminal records. From the procedural data, information about the participants' prior criminal record (i.e., crimes for which they have been convicted in the past) was also accessed. The SSPI-2 was coded by the first and second authors of the article, and only one of the coders was aware of the individual’s SVR-20 results. It is important to point out that the SVR-20 was filled out in the initial phase of the assessment process and that only later was the SSPI-2 scored. As previously mentioned, there was perfect agreement between the two SSPI-2 coders, which counters the idea that SSPI-2 scores may have been affected by knowledge of SVR-20 scores.

## Results

### Identification of Pedophilic Interests by SSPI-2

In our sample, 17 of the 170 (10%) men convicted of sexual offenses against children were considered to be likely to have preferential pedophilic sexual interests because they scored 4 or 5 on the SSPI-2. The average SSPI-2 score for convicted individuals in the present study was 1.76 (SD = 1.23).

### Comparison with SSPI-2 Development Sample

Comparing our results regarding the specific items of the scale with the results obtained in the original study by Seto et al. (2017a) utilizing a chi-square analysis, we found lower proportions regarding the following items: “Any boy victim under the age of 15”, “Multiple child victims under the age of 15”, “Any child victim under the age of 12” and “Any extrafamilial child victims under the age of 15”. As for the item “Any possession of child pornography” the proportions we found were higher compared with the original study. These differences were statistically significant (Table 2).

**Table 2.** Item Analysis: Mean, SD, Current Study Results Regarding the Specific items of the Scale versus Development Study Results.

Items	Current study (*N* = 170)	Development study (*N* = 1800)	*X* ^*2*^	*p*	*Cramer’s V*
		(Seto et al., 2017a)			
	(%)	(%)			
Male victim	16.5	30	13.84	<0.001	0.08
Multiple victims	24.7	51	43.18	<0.001	0.14
Victim below 12	54.1	73	27.31	<0.001	0.11
Extrafamilial victim	60.6	80	34.67	<0.001	0.13
Child sexual exploitation material	20.6	10	18.09	<0.001	0.10

### Inter-Item Correlations and Item-Total Correlations of SSPI-2

To better understand the properties of the SSPI-2, we calculated Spearman correlations among the items and then for each item and the total score. We used Karl Pearson’s (1904) definition to define the strength of an association, being classified as “high” when it was between 0.75 and 1; “considerable” when it was between 0.50 and 0.75; “moderate” between 0.25 and 0.50 and “low” between zero and 0.25.

There were moderate statistically significant correlations between the item “male victim” and the item “multiple victims”. There were low statistically significant correlations between the item “male victim” and the item “child pornography”. There were moderate statistically significant correlations between the item “multiple victims” and the item “child pornography”, and considerable statistically significant correlations between the item “extrafamilial victim” and the item “child pornography”.

As expected, the SSPI-2 total score was significantly and positively correlated with all five items.

### Convergent and Construct Validity

Spearman correlations were calculated for the scores obtained on the SSPI-2 and the scores for the “sexual deviance” item on the SVR-20. As expected, a statistically significant, moderate, and positive association was identified between the SSPI-2 total score and the sexual deviance item of the SVR-20, *r*_*s*_ = .31, *p* < .001 (Table 3).

**Table 3.** Intercorrelations Among SSPI-2 Items and the “sexual Deviance” item of SVR-20 for the Validation Sample (*N* = 170).

Itens	Male victim	Multiple victims	Victim below 12	Extrafamilial victim	CSEM	SSPI-2 total
Male victim	1					
Multiple victims	.30*	1				
Victim below 12	.12	.14	1			
Extrafamilial victim	.07	.04	−.04	1		
CSEM	.21**	.38*	−.03	.32*	1	
SSPI-2 total	.51*	.60*	.50*	.54*	.59*	1
“Sexual deviance” item of SVR-20	.30*	.50*	.14	.30*	.64*	.31*
SRP-SF	−.02	−.04	.02	−.22***	−.09	−.18
LIF	.03	−.06	.07	−.28**	−.17	−.10
ANT	.16	.01	.17	−.24***	−.13	−.11

*Note.* CSEM = child sexual exploitation material. Spearman’s rank-order correlations between SSPI-2 items, and items-total, and for the SSPI-2 and “sexual deviance”. Pearson’s correlations between SSPI-2 total and SRP-SF, and between SSPI-2 total and SRP-SF facets (LIF and ANT). Point-biserial correlation between SSPI-2 items and SRP-SF, as well as ANT and LIF facets. We used Karl Pearson’s (1904) definition to define the strength of an association, being classified as “high” when it was between 0.75 and 1; “considerable” when it was between 0.50 and 0.75; “moderate” between 0.25 and 0.50 and “low” between zero and 0.25.

*p < .001; **p < .01; ***p < .05.

A binary logistic regression analysis was performed with the total SSPI-2 score to predict the “sexual deviance” item score. The results indicated a highly significant association, with χ^2^(1) = 70.125, *p* < .001, and a pseudo *R*^2^ Nagelkerke of .70, signifying substantial explanatory power. Specifically, for each unit increase in SSPI-2, there was a 11.8 (OR = 11.8; IC 95% = 4.35 – 32.03) times greater likelihood that the individual would show “sexual deviance” on the SVR-20 item.

Receiver operator characteristic (ROC; Swets, 1988) analysis were performed to examine the discriminative capacity of the SSPI-2 in relation to the absence or presence of “sexual deviance” on the SVR-20 item. The AUC was .95 (SE = .03; *p* < .001; 95% CI = .91 – 1), indicating excellent discriminative ability.

To illustrate these results, we made a cross-tabulation between the SSPI-2 scores and “sexual deviance” (Table 4). No individual with a score of zero or 1 on the SSPI-2 was classified as having “sexual deviance” on the SVR-20, and only one individual with a score of 4 or 5 was scored “absent” on the SVR-20 item.

**Table 4.** Cross-tabulation Between the SSPI-2 Totals and “sexual Deviance” item on the SVR-20.

SSPI-2 score	SVR-20			
	Absent	Possible presence	Presence	No information
0	21	0	0	3
1	43	4	0	5
2	45	4	2	6
3	15	2	2	1
4	0	1	9	1
5	1	0	5	0

ROC curves were used to examine the discriminative capacity of the SSPI-2 in relation to the presence of previous convictions for sexual crimes against children under 15 years of age (coded as no or yes). The results demonstrated a statistically significant curve (*AUC* = .78; *SE* = .08; *p* = .004; 95% CI = .62 - .94), demonstrating that if randomly chosen, 78% of participants who had previous convictions for sexual crimes against children will have higher SSPI-2 scores than those than those who have no prior convictions.

Next, the analysis was conducted between the SSPI-2 and having three or more child victims, using the threshold indicated in Blanchard et al. (2001). The results demonstrated a statistically significant curve (*AUC* = .78; *SE* = .06; *p* = .021; 95% CI = .65 - .91), indicating that if randomly chosen, 78% of men who have sexual abused three or more children will have higher scores than those who victimize one or two child victims.

### Divergent Validity

Pearson correlations were calculated to assess divergent validity (Table 3). The SSPI-2 score was negatively but nonsignificantly correlated with the SRP-SF total score, *r* = −.18, *p* = .06. The same analyses were performed to assess the dimensions of the SRP-SF with adequate Cronbach’s alpha values for further analysis (i.e., equal to or greater than .70; Marôco & Garcia-Marques, 2006). The correlations with the Antisocial (*r* = −.11, *p* = .25) and Lifestyle facets (*r* = −.10, *p* = .30) were negative, nonsignificant, and weak. Also, point-biserial correlations were calculated for SSPI-2 items and SRP-SF scores, as well as the Antisocial and Lifestyle facets. The correlations were non-significant except for the “extrafamilial victim” item, which showed a moderate, negative, and significant correlation with the Lifestyle facet, and a low, positive, and significant correlation with the Antisocial facet and the SRP-SF total.

We found that only two individuals in our sample had previously committed sexual offenses against adults, and their total scores on the SSPI-2 were zero and 1. Because there were only two such individuals, we do not report the association between SSPI-2 scores and sexual offending against adults.

Finally, we conducted ROC curves between the SSPI-2 and having prior nonsexual offenses (categorized as no or yes). The results demonstrated a non-statistically significant association between the variables (*AUC* = .52; *SE* = .05; *p* = .72; *95% CI* = 0.41 – 0.63).

## Discussion

The results of this study provided evidence of both convergent and divergent validity of the SSPI-2, with positive associations with the SVR-20 sexual deviance item, prior sexual convictions against children, and having 3 or more child victims, and nonsignificant associations with self-reported psychopathy and prior nonsexual crime history. Only 2 individuals (1.2%) out of the 170 in the sample had a history of sexual offenses against adults as well, and their SSPI-2 scores were zero and 1, indicating very low scores on pedophilic sexual interests for individuals who also committed sexual offenses against adults.

Further, 10% (*n* = 17) of the participants were deemed to have pedophilic sexual interests based on having a score of 4 or 5 on the SSPI-2. These values are below estimates that 20%–50% of men who have sexually offended against children meet criteria for pedophilic disorder (Gerwinn et al., 2018; Schmidt et al., 2013; Seto, 2009, 2018, 2019; Walker & Panfil, 2016). The mean score and standard deviation on the SSPI-2 in this study were also lower than in the original study, with significant differences on the 5 items (Seto et al., 2017a). A possible explanation for these differences is the fact that our population was recruited in correctional settings, rather than a clinical setting where the clinical referral may result in part because of suspected pedophilic disorder. Another possible explanation could be that our data was collected only through criminal files. This lack of direct access to self-report likely led to lower scores since the SSPI-2 is scored from both criminal records and individuals' self-reports of undocumented sexual offenses against children.

Although the SSPI-2 scores in this study were different than the original study sample, the sample seems to be representative of the Portuguese population of individuals convicted of sexual crimes against children. According to the Annual Report of Internal Security (Internal Security System, 2022), of the 97 individuals that were arrested in 2021 for sexual offenses against children, 46.9% had extrafamilial victims (60.6% in this study sample) and 83.1% had girl victims (compared to 82% in this study). Regarding the age of the victims, 47.4% were under the age of 11, which was the age cutoff used in the annual report, compared to 54% of the present sample having victims under the age of 12 (Annual Activity Report of the Commission for the Protection of Children and Young People, 2021). There is no official data on the number of cases with multiple child victims. Nevertheless, these results appear to be similar to the national reality and suggest that a considerable number of individuals who have sexually offended against children in Portugal do not exhibit pedophilic sexual interests.

Despite the lower proportions of present SSPI-2 items in the Portuguese sample compared to the development sample in Seto et al. (2017a), the relative likelihood of SSPI-2 items being scored as present was similar for two samples, with the highest proportions for extrafamilial victims and the lowest proportions for having boy victims. All these items are significantly correlated with the total SSPI-2 score in the present sample. Also, the total score of SSPI-2 and four of the five items of SSPI-2, except for the item “victim below 12”, were positively and significantly correlated with the “sexual deviance” item of the SVR-20. None of the individuals with a SSPI-2 score of zero or 1 were deemed to have sexual deviance present on the SVR-20, and only 1 of the individuals with a SSPI-2 score of 4 or 5 was coded as absent for sexual deviance on the SVR-20.”. SSPI-2 scores were associated positively and significantly with prior sexual convictions for sexual crimes against children and with having 3 or more child victims. These results support the construct and convergent validity of the SSPI-2 and the notion that pedophilic sexual interests can be assessed in different ways (Seto, 2013).

Previous research consistently demonstrates that correlations between various measures of sexual interest in children, such as self-report, viewing time and phallometric tests, tend to show only moderate levels of association (Schmidt et al., 2017). This suggests that the construct of sexual interest towards children may not be easily captured by a single measure; rather a multi-method approach is necessary and recommended for a comprehensive understanding. On the other hand, the lack of a significant correlation between the 'victim below 12′ item and the SVR-20 item may reflect the fact that having victims under age 12 is more closely associated with pedophilia than hebephilia, and both pedophilia and hebephilia might have been considered in the pedophilic disorder diagnoses on record, which contributed to the scoring the SVR-20 item. Though the definition of pedophilia in the DSM-5-TR specifically refers to prepubescent children, the criterion also specifies children up to age 13, where children aged 12 or 13 may in fact be pubescent in appearance. This would attenuate the association for this item, but not the other SSPI-2 items, which tap into both pedophilia and hebephilia as well (Stephens et al., 2019).

These results indicate that the SSPI-2 may serve as a valuable tool to assess pedophilic sexual interests when phallometric testing is not feasible, as in the Portuguese context. The SSPI-2 is useful because it can organize information about child sexual victims from self-report and criminal records. Moreover, the SSPI-2 is specific to sexual offending, since its scores were nonsignificantly (and negatively) associated with self-reported psychopathy. This finding is in line with prior studies conducted using the SSPI (Eher et al., 2015; Kingston et al., 2007). Furthermore, considering that the Lifestyle facet is associated with impulsive and reckless behaviors, the existence of a significant negative correlation between it and the “extrafamilial victim” item of the SSPI-2 suggests that these crimes may be more planned and less impulsive (Cohen et al., 2002a; 2002b). A study conducted by Sousa et al. (2023) identified that individuals who committed sexual offenses against extrafamilial children exhibited lower scores on the Lifestyle facet compared to those who offended intrafamilial children, although the differences did not reach statistical significance (Sousa et al., 2023).

### Limitations

This study had several limitations. First, the sample was not large, which precluded sensitivity analyses, for example, comparing those who got a prison sentence with those who got community sentences. Second, the SSPI-2 was scored using file information only, which would have suppressed these scores to some unknown degree because self-reported child victims were not included in the scoring because participants were not asked about undetected child victims when the SSPI-2 was scored. Though self-reported child victims might be mentioned in court documents, we think it is unlikely since the participants may have been concerned about admitting to undetected crimes and thereby affecting their sentencing.

Another limitation of this study was the inability to calculate inter-rater reliability for the SVR-20 since we did not have two investigators scoring the SVR-20 for the same participants in order to perform this comparison. This may limit the generalizability of the results and the interpretation of the data related to the SVR-20. The same limitation applies to the file coding, which was conducted by a single coder and therefore inter-rater reliability was not estimated. Another limitation related to the SVR-20 is that the scoring of this item was based not only on self-reported sexual interest in children, but clinical diagnoses of pedophilic disorder made by a psychologist or psychiatrist, where the inter-rater reliability of these diagnoses were not known.

A possible limitation is that there was overlap in the scoring of the SSPI-2 and SVR-20, where knowledge of the SVR-20 scores could have contaminated the scoring of the SSPI-2 by one of the two raters of the SSPI-2. This would inflate the association between SSPI-2 and SVR-20 scores. The risk of this confound is mitigated by the fact that the SSPI-2 was objectively scored from file information about criminal history, and the perfect agreement in SSPI-2 scores between the two coders (where the second coder was not aware of the SVR-20 scoring).

### Conclusions

The results suggest the SSPI-2 holds promise as a screening measure to identify individuals with a greater likelihood of showing greater sexual arousal to children than to adults, and thus a greater likelihood of pedophilia or hebephilia. This would be valuable in terms of suggesting greater risk of sexual recidivism (given the results of Seto et al., 2017b; Faitakis et al., 2023) and identifying an important treatment and supervision target. The SSPI-2 could play a valuable role in the assessment and management of individuals who have sexually offended against children in Portugal. Consistent with Stephens et al. (2019), we reinforce that caution is needed when using the SSPI-2 and it should not be used by itself, being important to include other elements for the assessment (e.g., self-report, any objective testing).

Future studies, in the Portuguese context should carry out a more careful selection of the sample, focusing on those with victims aged 13 or lower (and thus below the legal age of consent in Portugal) and consistent with the age suggestion in the DSM-5-TR criteria for pedophilic disorder. In this study sample, however, only 3 individuals had 14 year old victims; the rest of the sample all had child victims aged 13 or younger. It would also be helpful to look at the associations between SSPI-2 scores and self-reported interest in children and pedophilic disorder diagnosis in diverse, international samples. Finally, research comparing the predictive validities of the SSPI-2, self-reported interest in children, and pedophilic disorder diagnosis in international samples would be valuable.

## Note

1. The Portuguese Criminal Code (2023) considers as pornographic any material that, for sexual purposes, depicts minors (under the age of 18) engaging in real or simulated sexually explicit behavior, or contains any representation of their sexual organs or any other part of their body.
